# Mendelian randomization study of the relationship between blood and urine biomarkers and schizophrenia in the UK Biobank cohort

**DOI:** 10.1038/s43856-024-00467-1

**Published:** 2024-03-07

**Authors:** Bolun Cheng, Yunfeng Bai, Li Liu, Peilin Meng, Shiqiang Cheng, Xuena Yang, Chuyu Pan, Wenming Wei, Huan Liu, Yumeng Jia, Yan Wen, Feng Zhang

**Affiliations:** 1 Key Laboratory of Trace Elements and Endemic Diseases (Xi’an Jiaotong University), National Health and Family Planning Commission, 710061 Xi’an, China; 2 Key Laboratory of Environment and Genes Related to Diseases (Xi’an Jiaotong University), Ministry of Education, 710061 Xi’an, China; 3 Collaborative Innovation Center of Endemic Disease and Health Promotion for Silk Road Region, School of Public Health, Health Science Center, Xi’an Jiaotong University, 710061 Xi’an, China; 4 School of Public Health, Shaanxi University of Chinese Medicine, 712046 Xianyang, China

**Keywords:** Schizophrenia, Diagnostic markers

## Abstract

**Background:**

The identification of suitable biomarkers is of crucial clinical importance for the early diagnosis of treatment-resistant schizophrenia (TRS). This study aims to comprehensively analyze the association between TRS and blood and urine biomarkers.

**Methods:**

Candidate TRS-related single nucleotide polymorphisms (SNPs) were obtained from a recent genome-wide association study. The UK Biobank cohort, comprising 376,807 subjects with blood and urine biomarker testing data, was used to calculate the polygenic risk score (PRS) for TRS. Pearson correlation analyses were performed to evaluate the correlation between TRS PRS and each of the biomarkers, using calculated TRS PRS as the instrumental variables. Bidirectional two-sample Mendelian randomization (MR) was used to assess potential causal associations between candidate biomarkers with TRS.

**Results:**

Here we identify a significant association between TRS PRS and phosphate (*r* = 0.007, *P* = 1.96 × 10^−4^). Sex subgroup analyses identify seven and three candidate biomarkers associated with TRS PRS in male and female participants, respectively. For example, total protein and phosphate for males, creatinine and phosphate for females. Bidirectional two-sample MR analyses indicate that TRS is negatively associated with cholesterol (estimate = −0.363, *P*  = 0.008). Conversely, TRS is positively associated with total protein (estimate = 0.137, *P*  = 0.027), mean corpuscular volume (estimate = 0.032, *P*  = 2.25 × 10^−5^), and mean corpuscular hemoglobin (estimate = 0.018, *P*  = 0.007).

**Conclusions:**

Our findings provide insights into the roles of blood and urine biomarkers in the early detection and treatment of TRS.

## Plain language summary

People with schizophrenia experience periods of time during which they misperceive reality. Some people with schizophrenia do not respond well to the usual drugs that are used to relieve their symptoms. This type of schizophrenia is known as treatment-resistant schizophrenia (TRS). We looked at differences in the genes (inherited characteristics), blood and urine of a group of people in the UK with schizophrenia to see if people with TRS have particular characteristics that would enable them to be distinguished from patients with schizophrenia who tend to respond to usual treatment. We found several differences in the blood that could be used to predict which people might get TRS, including some that were specific to men or women. These discoveries are important because they can help doctors identify people who are more likely to develop TRS earlier, enabling them to avoid using treatments that might not work well for them.

## Abstract (web summary)

Cheng, Bai et al. uncover associations between 63 blood and urine biomarkers and treatment-resistant schizophrenia using data from the UK Biobank. They find correlations between some blood and urine biomarkers and treatment resistant schizophrenia polygenic risk scores.

## Introduction

Schizophrenia is a complex cognitive and behavioral syndrome that seriously affects the quality of life. It is characterized by emotional, cognitive, perceptual, and thought disorders^1,2^. Some individuals with schizophrenia experience treatment-resistant symptoms characterized by severe dysfunction in which symptoms do not completely respond to at least two first-line antipsychotic drugs^3^. Treatment-resistant schizophrenia (TRS) is a complex clinical condition that affects approximately 30% of people living with schizophrenia^4^. Studies suggest that TRS may have a higher heritability compared to schizophrenia, indicating that TRS may be a more familial phenotype and distinguishable from non-TRS cases based on its genetic underpinnings^5^. Patients with TRS have poorer prognosis and worse functional outcomes compared to patients with other severe psychiatric disorders^6^. By elucidating the genetic predisposition to specific biomarkers associated with TRS, it may be possible to develop more accurate predictive models for identifying individuals at risk of developing TRS. This could ultimately lead to earlier interventions and improved outcomes.

Peripheral blood and urine biomarkers are frequently measured to diagnose and evaluate chronic disease conditions^7^. Many biochemical indicators in peripheral blood and urine have been found to be abnormal in patients with mental diseases, such as schizophrenia^8^, major depressive disorder^9,10^, autism spectrum disorder^11^, and anxiety^12^. A recent large observational study demonstrated that higher concentrations of tau protein in peripheral blood were associated with cognitive degeneration in patients with Alzheimer’s disease (AD)^13^. It is noteworthy that peripheral blood biomarkers hold promise as a substitute for the central nervous system in characterizing psychiatric disorders, although their role has yet to be widely applied in clinical practice. Urine is also a convenient and appropriate substance for use in diagnostic or predictive tests for disease, as subtle changes in urine are accumulated in the blood and are unaffected by homeostasis mechanisms^14^.

Early detection and diagnosis of schizophrenia and TRS are crucial for their prevention and treatment. Screening for suitable biomarkers is essential for early diagnosis. In the past decade, considerable efforts have been made to identify reliable biomarkers for the early detection of schizophrenia. Blood biomarkers are considered as a viable option because the dysregulation of epigenetic patterns, gene expression, metabolic and inflammatory molecules in peripheral blood have unique patterns in individuals with schizophrenia^15^. Abnormal metabolic, immune, and hormonal alterations have been found in the blood of patients with schizophrenia^16^, such as inflammatory biomarkers (C-reactive protein and interleukins)^17^ and neurotrophic biomarkers (BDNF protein)^18^. Additionally, measurements of urinary bufotenine levels in patients with schizophrenia and healthy controls without psychotic symptoms found that increased urine levels of the endogeneous psychogenic molecule bufotenine may play a vital role in schizophrenia^19^. Studies have also demonstrated the genetic basis of serum and urine biomarkers and their causal effects on psychiatric disorders^7^. Although the information contained in blood and urine is not entirely comprehensive, the observed changes may still be used for diagnostic and monitoring purposes.

The polygenic risk score (PRS) is a powerful tool for predicting an individual’s genetic inclination and the severity of mental disorders^20^. This is achieved by weighting and calculating the effect size of SNPs^20^. The PRS has been widely used in exploring the correlations between the genetic susceptibility of multiple diseases in phenome-wide association studies^21,22^.

In this study, we comprehensively analyze the correlation between blood and urine biomarkers with TRS. The TRS PRS are calculated using genotype data from the UK Biobank (UKB) cohort. Pearson correlation analyses are performed to investigate the correlation between each blood or urine biomarker and TRS. Bidirectional two-sample Mendelian randomization analyses are performed to validate the candidate correlations between biomarkers and TRS. In the analysis of TRS PRS-associated blood and urine biomarkers across the total UKB sample, a significant association is found with phosphate at the Bonferroni correction threshold. Six biomarkers exhibit candidate association signals with TRS PRS. Bidirectional Mendelian randomization analyses reveal significant associations between TRS and various biomarkers. TRS is positively associated with mean corpuscular volume but negatively associated with total protein according to IVW MR analyses. MR-Egger analyses suggest an opposite causal direction between TRS and total protein and reveal a causal relationship between TRS and cholesterol. Weighted median MR analyses confirm the associations observed in IVW analyses and reveal additional associations with lymphocyte count and mean corpuscular hemoglobin. Our study provides insight into the application of polygenic risk scores and highlights the importance of blood and urine biomarkers in the early diagnosis of TRS.

## Methods

### Biomarker phenotypes in serum and urine in UKB cohort

The phenotypic and genotypic data used in this study were obtained from the UKB, which conducted a large prospective cohort study from 2006 to 2010^23^. The UKB performed laboratory testing of commonly measured biomarkers in serum (Category 100080) and urine (Category 100083) on a cohort with extensive phenotype and genome-wide genotype data, including the unrelated individuals in this study^23^. Health-related records of each participant, including age and sex, were collected through either a screenshot questionnaire or verbal interview within the assessment center. The urine assays category contains information on the assays that have been performed on the UKB urine samples (https://biobank.ndph.ox.ac.uk/showcase/label.cgi?id=100083), while the blood assays category contains information on the assays that have been performed on the UKB blood samples and their results (https://biobank.ndph.ox.ac.uk/showcase/label.cgi?id=100080), including blood count (Category 100081) and blood biochemistry (Category 17518). Blood count contains results of hematological assays that were performed on whole blood before further processing, including data on acquisition time and number of freeze-thaw cycles, such as basophils, eosinophils, monocytes, and neutrophils. Blood biochemistry contains a range of key biochemistry markers that were measured in blood samples collected at recruitment and at repeat assessment. The ethical approval of UKB was granted by the National Health Service National Research Ethics Service (reference 11/NW/0382). All participants gave informed consent for participation in the UKB. Permission to access and analyze UKB data was approved under UKB project 46478. The need to seek additional ethical approval for our study from our University was waived because the study involved the secondary use of data.

### UK Biobank genotyping, imputation and quality control

Genome-wide genotyping was performed in UKB individuals using either the Affymetrix UKB Axiom array or Affymetrix UK BiLEVE Axiom, which included 812,428 SNPs. Imputation was conducted using IMPUTE2 with the reference panel of the UK10K projects, Haplotype Reference Consortium, and 1000 Genomes^23^. For quality control on the genotype data, the UKB excluded the SNPs with INFO < 0.9, Hardy–Weinberg equilibrium (HWE) testing *p* values < 0.0001, minor allele frequencies (MAF) < 0.01 and genotyping call rate <95%. A total of 488,377 individuals and 805,426 SNPs were kept after applying quality control (QC). After removing individuals who reported inconsistencies between self-reported sex and genetic sex, as well as those with missing covariate information, 376,807 individuals of white British ancestry subset (UK Biobank field ID: 21000)^23^ were retained for further analysis^23^.

### Polygenic risk score datasets of treatment resistance in schizophrenia

The GWAS summary statistics of TRS were obtained from Pardiñas et al.^4^. The study analyzed the GWAS of TRS based on a sample size of 10,501 individuals with TRS and 20,325 individuals without TRS. All individuals with TRS were administered clozapine in the UK, in accordance with the National Institute for Health and Care Excellence guidelines for TRS, after the failure of at least two trials of antipsychotics^24^. Due to the involvement of different datasets^25^ and genotyping arrays^26^ in this analysis, the processing of TRS and non-TRS GWAS samples was conducted separately on data generated from the original studies. Imputations were performed using the SHAPEIT/IMPUTE2 workflow^27,28^. The combination results of non-TRS GWAS by the Psychiatric Genomics Consortium were analyzed using the fixed-effects program in METAL^29^. The combined samples with SNPs called in <20,000 and any strand ambiguous markers with minor allele frequency (MAF) ≥ 40% were removed. Detailed information on genotyping, imputation, quality control, and statistical analysis is available in the eMethods by Pardiñas et al.^4^. To generate association statistics that accurately reflect differences between TRS and non-TRS groups, Pardinas et al. utilized the test for interaction proposed by Altman and Bland^30^. This test is similar to a fixed-effect test for moderators in the meta-analytic setting^4^.

### PRS calculation of treatment resistance in schizophrenia in UK Biobank cohort

The clumping algorithm in PRSice-2 (version 2.3.5) was configured to identify any SNPs within 250 kb in linkage disequilibrium (LD) with an *r*^2^ threshold greater than 0.1 and ‘clump’ them together to be represented by the most significantly associated SNP within each LD block, known as the index SNP^31^. PRSice-2 was employed to generate PRS for TRS, utilizing recent large GWAS summary statistics of TRS^4^. Significance values and effect sizes from the UKB cohort were used to generate the best PRS model. In brief, the PRS for each individual was calculated by summing the effect sizes from all the SNPs included in the best model. To generate the best-fit PRS of TRS, clozapine use (UKB Data-Field 20003) was used as a proxy measure of TRS, with the top 10 principle components (PC) of population structure, age, and sex included as covariates. The PRS analysis was conducted, comparing 45 participants using clozapine to a larger group of 376,762 participants not using clozapine, irrespective of their schizophrenia diagnosis status. The best model was derived by testing the inclusion of SNPs (401–89,618 SNPs) from a range of *P* value thresholds from 5 × 10^−8^ to 1 with an incremental interval of 0.0005 (--interval 0.0005 --lower 5e−08), to determine which threshold gave the largest Nagelkerke’s *R*^2^ value. These SNPs were then used to generate PRS for each individual in UKB cohorts. The performance of the best model for the selection of SNP markers was evaluated by the area under the receiver operating characteristic (ROC) curve (AUC) using the pROC R package^32^.

### Statistics and reproducibility

The PRS of TRS were initially adjusted for the PC1–PC10 using linear regression models. The rstandard function was then utilized to calculate the standardized residual of PRS, which is obtained by dividing the residual by its standard deviation (SD). The measurements of 59 blood and 4 urine biomarkers were adjusted for potential confounding variables, including sex (excluded in sex stratification analysis) and age, through linear regression models. The aim of this model was to extract the residual biomarker measurements and eliminate the effects of these covariates from subsequent Pearson correlation analysis. The resulting residuals were then utilized as the phenotypic values of biomarkers for the subsequent analyses. Pearson correlation analyses were conducted to investigate the correlation between each biomarker and TRS phenotype, with standardized PRS calculated as instrumental variables. Males and females were divided into separate groups to facilitate a better analysis of differences between sex stratification groups. The significant *P* value thresholds of Pearson correlation should be 7.94 × 10^−4^ (0.05/63 independent biomarkers) after strict Bonferroni correction. All statistical analyses, including AUC, linear regression, and Pearson correlation analyses, were performed using R software (version R 4.3.0).

### Bidirectional two-sample Mendelian randomization analyses

To validate potential causal associations between blood and urine biomarkers with TRS, we performed bidirectional two-sample MR analyses for significantly correlated blood and urine biomarkers. The present study utilizes candidate blood and urine biomarkers derived from GWAS summary statistics, which are publicly available at https://gwas.mrcieu.ac.uk/. The GWAS summary statistics dataset of TRS was obtained from the GWAS data by Pardiñas et al.^4^. Initially, we extracted SNPs that showed genome-wide statistical significance in association with exposure phenotypes. Subsequently, two-sample MR analyses were carried out using R (version 4.3.0) and the TwoSampleMR package^33^. Exposure and outcome GWAS summary statistics were harmonized by aligning summary statistics to infer positive strand alleles using allele frequencies for palindromes to ensure that the effect of an SNP on the exposure, and the effect of that same SNP on the outcome, corresponds to the same allele.

We performed fixed-effects analysis of genetic instruments using inverse variance-weighted (IVW) MR^34^. The MR-Egger regression analysis and weighted median MR approach were utilized to evaluate the robustness of our findings^35,36^. To assess horizontal pleiotropy, we tested for the presence of statistically significant (*P*  <  0.05) heterogeneities in MR-Egger analyses using the Cochran *Q* statistic^37^. In assessing consistency and robustness, we sought estimates that substantially agreed in direction and magnitude (overlapping confidence intervals) across complementary MR methods.

### Reporting summary

Further information on research design is available in the Nature Portfolio Reporting Summary linked to this article.

## Results

### Descriptive characteristics of study participants

This cross-sectional study included a total of 376,807 participants aged between 39 and 73 years, with a mean age of 56.99 years and a standard deviation of 7.93 years. The study population consisted of 202,434 male and 174,373 female subjects. Biomarker test results from blood and urine assays were obtained from all 376,807 study participants and were utilized for further analysis. The detailed statistical information on serum and urine biomarkers in this study is presented in Supplementary Data 1.

### PRS Nagelkerke *R*^2^ and AUC

The PRS models suggest that a stricter cutoff may result in the missing of informative SNPs, while a looser cutoff may introduce noise by including SNPs with spurious TRS association (Supplementary Data 2). The best-fit PRS at a *P* value threshold = 0.0015 (*R*^2^ = 0.007) was utilized for downstream analysis (Supplementary Fig. 1A). A screening test is considered better than pure chance if the AUC value is >0.5^38^. Using the PRS of the best model as the predictor and the TRS participant group as the outcome, the AUC was 0.61 (95% CI: 0.48–0.74). With the threshold of PRS cutoff 0.252, the sensitivity (true positive rate, TPR) for TRS prediction is 52.17%, and the specificity (true negative rate, TFR) for TRS prediction is 78.26% (Supplementary Fig. 1B).

### TRS PRS-associated blood and urine biomarkers in total, male and female population

**Fig. 1:**
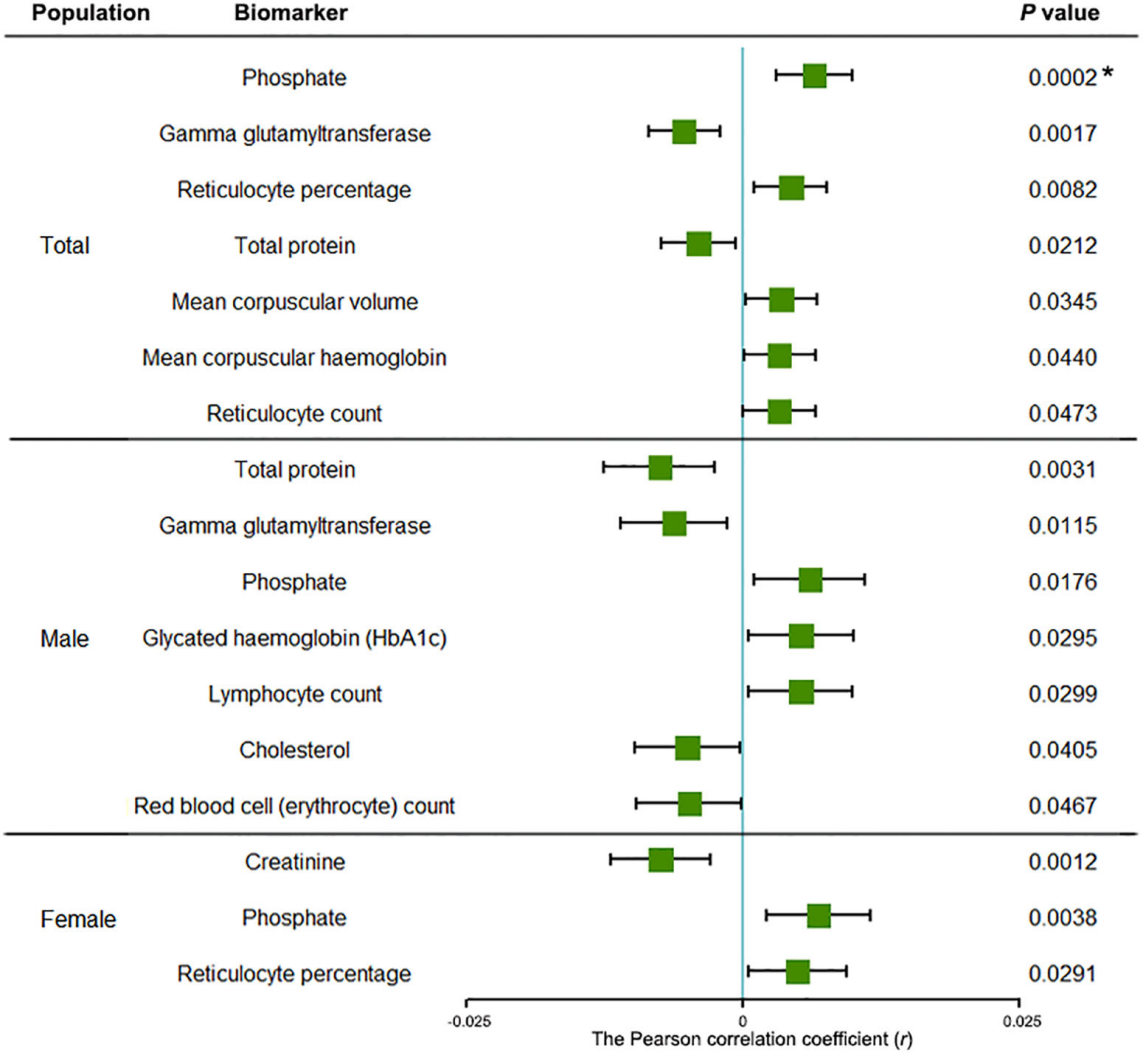
The significant correlations between biomarkers and treatment-resistant schizophrenia in total, male and female populations. The error bars indicate 95% confidence intervals (CIs); Dots indicate for specific Pearson correlation coefficient (*r*) of biomarkers; Horizontal lines represent 95% CI. An asterisk (*) indicates the biomarker that has reached the significant threshold of Bonferroni correction (7.94 × 10^−4^).

Upon analyzing the UKB total sample, we identified a significant association between TRS PRS and phosphate (*r* = 0.007, *P* = 1.96 × 10^−4^) at the Bonferroni correction threshold (Fig. 1). Furthermore, we identified six biomarkers that have candidate association signals with TRS PRS, such as gamma-glutamyltransferase (*r* = −0.005, *P* = 0.002), reticulocyte percentage (*r* = 0.004, *P* = 0.008), and total protein (*r* = −0.004, *P* = 0.021). Detailed information on Pearson correlation results for the total population is presented in Supplementary Data 3. Upon testing for association with TRS PRS in male and female participants, no blood or urine biomarker exhibited statistical significance after strict Bonferroni correction (Fig. 1). However, in the male participants of the UKB cohort, we identified seven biomarkers that were potentially associated with TRS PRS at the general *P* threshold (Supplementary Data 4), such as total protein (*r* = −0.008, *P* = 0.003), gamma-glutamyltransferase (*r* = −0.006, *P* = 0.012), and phosphate (*r* = 0.006, *P* = 0.018). In the female participants, we observed three candidate biomarkers for TRS PRS (Supplementary Data 5), including creatinine (*r* = −0.007, *P* = 0.001), phosphate (*r* = 0.007, *P* = 0.004), and reticulocyte percentage (*r* = 0.005, *P* = 0.029).

### Bidirectional Mendelian randomization analyses

Table 1 presents fundamental information on the GWAS summary data used in bidirectional two-sample MR. The sample size, number of significant SNPs, and heritability are provided for each trait analyzed. The traits investigated in this study include TRS, phosphate, gamma glutamyltransferase, reticulocyte percentage, total protein, mean corpuscular volume, mean corpuscular hemoglobin, reticulocyte count, glycated hemoglobin (HbA1c), lymphocyte count, cholesterol, red blood cell (erythrocyte) count, and creatinine. The number of genome-wide significant independent loci for each trait is indicated by SNPs (*n*), with SNPs within 10,000 kilobase pairs and *R*^2^ ≥ 0.001 removed.

**Table 1:** The basic information of GWAS summary data used in two-sample Mendelian randomization validation

Traits (Ref code)	Sample size (*n*)	Significant threshold	SNPs (*n*)	Heritability (SE)
TRS (/)	30,826	5 × 10^−6^	9	0.013 (0.006)
Phosphate (met-a-364)	7789	5 × 10^−5^	19	0.107 (0.010)
Gamma-glutamyltransferase (ukb-e-30730_CSA)	8422	5 × 10^−5^	93	0.197 (0.025)
Reticulocyte percentage (ukb-d-30240_irnt)	344,728	5 × 10^−8^	288	0.179 (0.014)
Total protein (bbj-a-56)	113,509	5 × 10^−8^	36	0.137 (0.009)
Mean corpuscular volume (ukb-d-30040_irnt)	350,473	5 × 10^−8^	378	0.234 (0.026)
Mean corpuscular hemoglobin (ukb-d-30050_irnt)	350,472	5 × 10^−8^	366	0.229 (0.028)
Reticulocyte count (ukb-d-30250_irnt)	344,729	5 × 10^−8^	291	0.180 (0.013)
Glycated hemoglobin (HbA1c) (ukb-e-30750_CSA)	8329	5 × 10^−8^	5	0.188 (0.016)
Lymphocyte count (ukb-d-30120_irnt)	349,856	5 × 10^−8^	343	0.171 (0.011)
Cholesterol (ieu-a-301)	187,365	5 × 10^−8^	88	0.222 (0.028)
Red blood cell (erythrocyte) count (ukb-d-30010_irnt)	350,475	5 × 10^−8^	358	0.196 (0.016)
Creatinine (ieu-a-1105)	133,814	5 × 10^−8^	47	0.094 (0.007)

SNPs (*n*) indicates the number of genome-wide significant independent loci (we removed SNPs within 10,000 kilobase pairs and *R*^2^ ≥ 0.001) under a significant threshold.

*TRS* treatment-resistant schizophrenia, *Ref code* reference code.

**Fig. 2:**
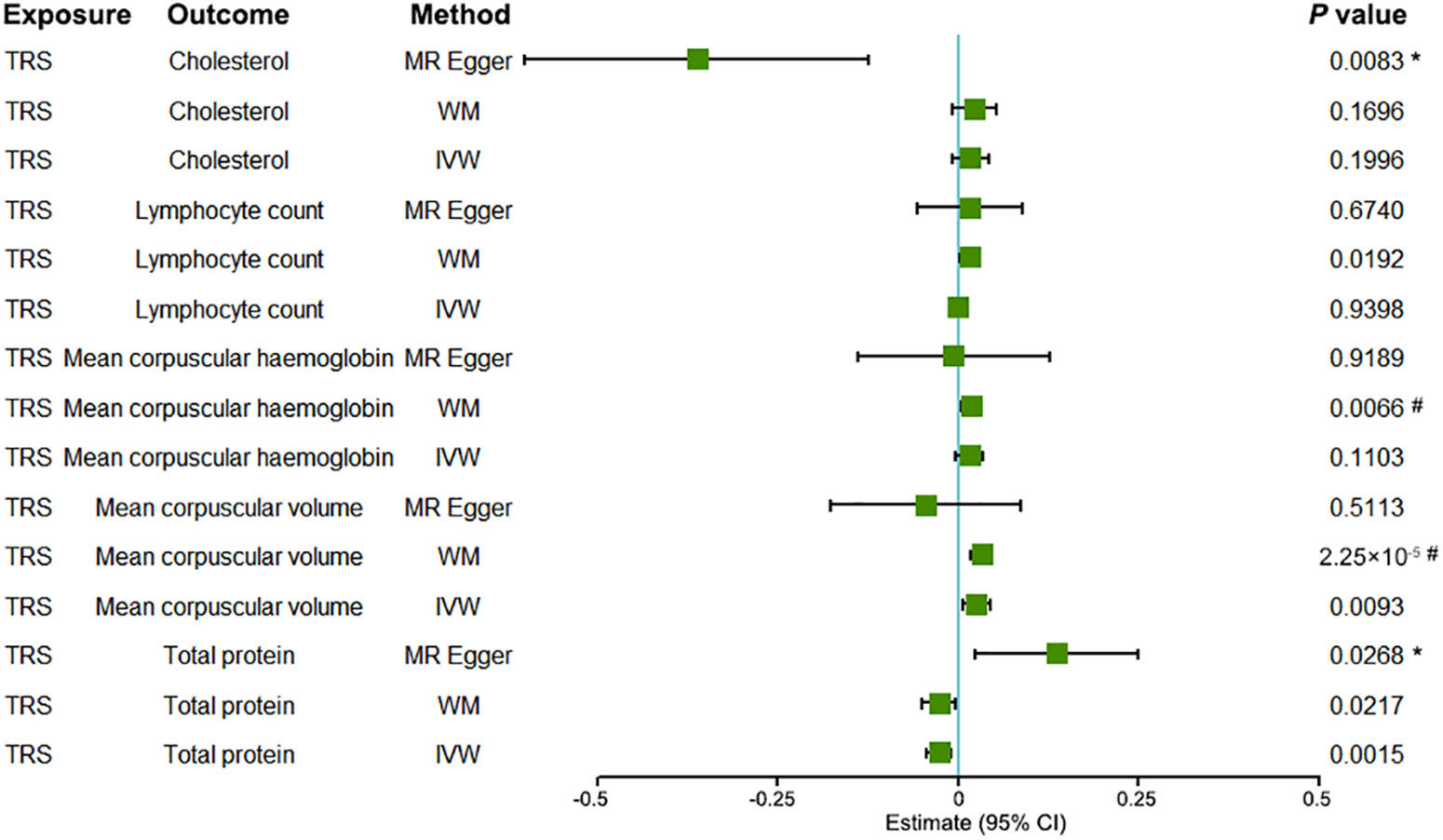
Significant MR results for the causal relationship between TRS and biomarkers. The error bars indicate 95% confidence intervals (CIs). An asterisk (*) indicates the presence of pleiotropy in this MR analysis, which results in more precise estimates from MR-Egger. A hashtag (#) indicates the presence of heterogeneity in this MR analysis, which results in more precise estimates from the weighted median. TRS treatment-resistant schizophrenia, IVW inverse variance weighted, WM weighted median, MR Mendelian randomization.

Figure 2 presents the significant bidirectional MR results for TRS and candidate biomarkers. The IVW MR analyses indicated that TRS was positively associated with mean corpuscular volume (estimate (SE) = 0.025 (0.010), *P* = 0.009), while TRS was negatively associated with total protein (estimate (SE) = −0.027 (0.009), *P*  = 0.002) (Fig. 2). In contrast, MR-Egger analyses showed an opposite causal direction between TRS and total protein (estimate (SE) = 0.137 (0.058), *P*  = 0.027). Notably, MR-Egger analyses demonstrated a causal relationship between TRS and cholesterol (estimate (SE) = −0.363 (0.122), *P*  = 0.008). The weighted median MR analyses showed the same causal direction as IVW analyses for the associations between mean corpuscular volume, total protein and TRS (Fig. 2). Additionally, the weighted median MR analysis showed a slightly weaker causal relationship between TRS and lymphocyte count (estimate (SE) = 0.015 (0.007), *P*  = 0.019), and mean corpuscular hemoglobin (estimate (SE) = 0.019 (0.007), *P*  = 0.007). Supplementary Data 6 provides detailed MR estimates of genetic instruments for TRS and candidate biomarkers based on Pearson correlation results.

To evaluate the potential for horizontal pleiotropy, we assessed heterogeneity and conducted sensitivity analyses that are more robust to pleiotropy, including weighted median MR and MR-Egger regression. Heterogeneity tests did not indicate heterogeneity in the IVW and MR-Egger estimates for the association of TRS with cholesterol, lymphocyte count, and total protein (*P* range, 0.089–0.960) (Supplementary Data 7). Notably, heterogeneity was observed in mean corpuscular volume, mean corpuscular hemoglobin, and TRS, so the weighted median method was more accurate for these two biomarkers^36^. The MR-Egger intercept analysis indicates that horizontal pleiotropy exists between TRS and cholesterol and total protein, indicating that the MR-Egger is more accurate in the MR analysis of TRS and these two biomarkers^36^ (Supplementary Data 8).

## Discussion

In this study, we conducted a large observational and genetic PRS analysis to comprehensively evaluate the associations between blood/urine biomarkers and TRS using the UKB cohort. We then performed bidirectional MR analysis to validate the candidate causal relationship. Our findings revealed several potential correlations in blood and urine biomarkers. Further analysis provided tentative evidence for sex differences in the characteristics between TRS with blood and urine biomarkers.

Currently, there is no objective biological measurement available for the diagnosis of TRS. Phosphate is an essential mineral for humans and plays numerous functions in the body. The blood level of phosphate is tightly regulated within a narrow range. Both hyperphosphatemia and hypophosphatemia can lead to the development of diseases^39^. The disturbed integrity of myelin and white matter, along with dysregulation of lipid metabolism, may be involved in schizophrenia pathophysiology^40^. Previous studies have reported increased high-energy phosphate metabolism in the basal ganglia of neuroleptic-naive schizophrenia patients using magnetic resonance spectroscopy (MRS)^41,42^. However, there are very few studies investigating the relationship between phosphate concentration and TRS in schizophrenia patients. Our study provides evidence for a correlation between phosphate and TRS, suggesting that blood phosphate may be a potential biomarker in TRS.

Previous studies have identified some biomarkers associated with TRS or schizophrenia, but not with TRS PRS in our study, such as bilirubin and creatinine. Bilirubin, the final product of heme metabolism in the body, is an endogenous antioxidant with an anti-inflammatory effect^43^. Unbound bilirubin has been studied as a promising molecule that could be used as a possible biological marker for schizophrenia^44^. In a prospective study, blood levels of unbound bilirubin were higher in patients with schizophrenia than in patients with bipolar disorder^45^. Serum creatinine is one of the most commonly measured products in clinical chemistry laboratories worldwide^46^ and has been linked with many neurodegenerative diseases^47^. The evidence linking schizophrenia and creatinine is primarily from observational studies. Researchers have identified multiple potential metabolite biomarkers of schizophrenia, such as reduced levels of essential polyunsaturated fatty acids and creatinine^48^. Additionally, an association with schizophrenia was also found in urine. Previous studies have shown that urinary creatinine concentrations were reduced in patients with schizophrenia compared with healthy controls^49,50^. These studies, together with our results, suggest that these biomarkers may not be influenced by individual genetic inclination.

Epidemiological data has revealed sex differences in the prevalence of schizophrenia^51^. Similarly, sex-specific blood biomarkers were observed in this study. Glycated hemoglobin (HbA1c), lymphocyte count, and red blood cell (erythrocyte) count showed potential correlation signals in male samples, while creatinine and reticulocyte percentage showed potential correlation signals in female samples. Sex-specific differences associated with age of onset, duration, and antipsychotic response in schizophrenia may be reflected in sex-related differences in the underlying molecular pathways^52^. Researchers have identified the structural and neurophysiological sex characteristics of schizophrenia by focusing on specific biomarkers^53^. A study found that males with schizophrenia had higher glycated hemoglobin (HbA1c), lower high-density lipoprotein, and an earlier age of onset compared to females^54^. Neurosteroids, including DHEA, DHEA-S, and pregnenolone, are involved in the pathophysiology of schizophrenia in male patients, but not in female patients^55^. Therefore, our results emphasize the importance of considering sex-specific differences in TRS research and highlight the clinical potential of these blood biomarkers for predicting TRS. Although the TRS PRS demonstrates potential as a tool for identifying individuals with an elevated predisposition to developing TRS, it is not a conclusive diagnostic measure. There may be instances where individuals with a high TRS-PRS do not ultimately develop TRS, while those with a low TRS-PRS may indeed manifest symptoms of TRS. Thus, it is crucial to recognize that TRS-PRS requires complementary clinical assessments for precise diagnosis and effective treatment.

Our bidirectional MR analyses demonstrated a negative association between TRS and cholesterol. In terms of metabolic alterations, Francesca et al. observed lower levels of high-density lipoprotein (HDL)-cholesterol among male treatment-resistant patients treated with clozapine^56^. The study selected a total of 33 patients previously diagnosed with TRS, who were prescribed clozapine followed by PP1M and PP3M when available, and the data showed a decrease in cholesterol^57^. These findings are consistent with the results of our MR analysis. Furthermore, we found that TRS was positively associated with red blood cell parameters, including mean corpuscular volume and mean corpuscular hemoglobin. Studies compared differences in mean corpuscular volume and mean corpuscular hemoglobin (MCH) in patients with schizophrenia, and found differences for all red cell parameters between study groups^58^. Age and sex may affect various erythrocyte parameters^58^. Another study investigated changes in various biochemical parameters in schizophrenia patients using clozapine and found that MCH and red blood cell count (RBC) levels were lower in the clozapine group compared with healthy volunteers^59^. Future studies should verify our results and further explore the biological confounding factors that could explain associations between red blood cell parameters and TRS.

This study comprehensively investigates the association between blood/urine biomarkers and TRS. Our sample size maximizes power for genetic analyses. This study also has some limitations. Firstly, the TRS PRSs were computed utilizing a subgroup of the UKB cohort consisting of individuals with white British ancestry, primarily of middle-aged demographics. Therefore, caution should be exercised when applying our results to young people and other ethnic populations. Secondly, the use of residuals may have impacted the size of the effects reported, and future studies should consider alternative methods to further validate our findings. The use of independent clinical specimens or cohorts to validate our findings and investigate the underlying biological mechanisms of the observed association between candidate blood and urine biomarkers with TRS will be necessary in future studies. Thirdly, the method and accuracy of the UKB biomarker measurement have potential implications for our results. Finally, due to the highly polygenic and pleiotropic nature of the genetic architecture of psychiatric phenotypes, the core MR assumptions are easily violated^60^. Pleiotropy will bias estimates by reintroducing confounding, and heterogeneity in the outcome will reduce the precision of our causal estimates, making it harder to identify a true causal effect should one exist^61^. Therefore, all analyses should be carefully considered and cautiously interpreted.

In conclusion, our study systematically analyzed the associations between TRS and blood/urine biomarkers using UKB individual-level traits and genotype data and TRS GWAS summary data. Our study highlights the sex-related differences in the underlying blood biomarkers, identifies the associations between TRS with phosphate and cholesterol, and may provide insights to reveal the roles of blood/urine biomarkers in the development of TRS. Our findings of potential blood biomarkers for TRS may be useful for diagnostic purposes, as well as for drug development and monitoring disease progression.

### Supplementary information

Supplementary Information

Description of Additional Supplementary Files

Supplementary Data 1

Supplementary Data 2

Supplementary Data 3

Supplementary Data 4

Supplementary Data 5

Supplementary Data 6

Supplementary Data 7

Supplementary Data 8

Reporting Summary

## Data Availability

Access to the UK Biobank data can be obtained by applying to the UK Biobank Access Management System, details are at https://www.ukbiobank.ac.uk/. We will return the derived data fields following UK Biobank policy; in due course, they will be available through the UK Biobank Access Management System. The source data underlying Figs. 1 and 2 are in Supplementary Data 3–5 and 6, respectively.

## Code availability

The R-based custom code for statistical analysis for this work is publicly available at https://github.com/DrBolunCheng/PRSMR/^62^.
