# Supplementary material for: Mendelian randomization study of the relationship between blood and urine biomarkers and schizophrenia in the UK Biobank cohort
Source: Commun Med (Lond). 2024 Mar 7;4:40. doi: 10.1038/s43856-024-00467-1 (PMC10920902; doi:10.1038/s43856-024-00467-1)
Supplement: Supplementary file 1 — Supplementary Information [file 43856_2024_467_MOESM1_ESM.pdf]

Supplementary Information

Mendelian randomization study of the relationship between blood and urine biomarkers and schizophrenia in the UK Biobank cohort

Bolun Cheng[1,2,3#], Yunfeng Bai[1,4#], Li Liu[1], Peilin Meng[1], Shiqiang Cheng[1], Xuena Yang[1], Chuyu Pan[1], Wenming Wei[1], Huan Liu[1,2,3], Yumeng Jia[1,2,3], Yan Wen[1,2,3], Feng Zhang[1,2,3*]

[1] Collaborative Innovation Center of Endemic Disease and Health Promotion for Silk Road Region, School of Public Health, Health Science Center, Xi'an Jiaotong University, 710061, Xi’an, China

[2] Key Laboratory of Trace Elements and Endemic Diseases (Xi'an Jiaotong University), National Health and Family Planning Commission, 710061, Xi’an, China

[3] Key Laboratory of Environment and Genes Related to Diseases (Xi'an Jiaotong University), Ministry of Education, 710061, Xi’an, China

[4] School of Public Health, Shaanxi University of Chinese Medicine, 712046, Xianyang, China

[#] These authors contributed equally

[*]Corresponding author: Feng Zhang

School of Public Health, Health Science Center, Xi'an Jiaotong University

Phone: 86-29-82655091

Email: fzhxjtu@mail.xjtu.edu.cn

No. 76 Yan Ta West Road, Xi’an, P. R. China, 710061

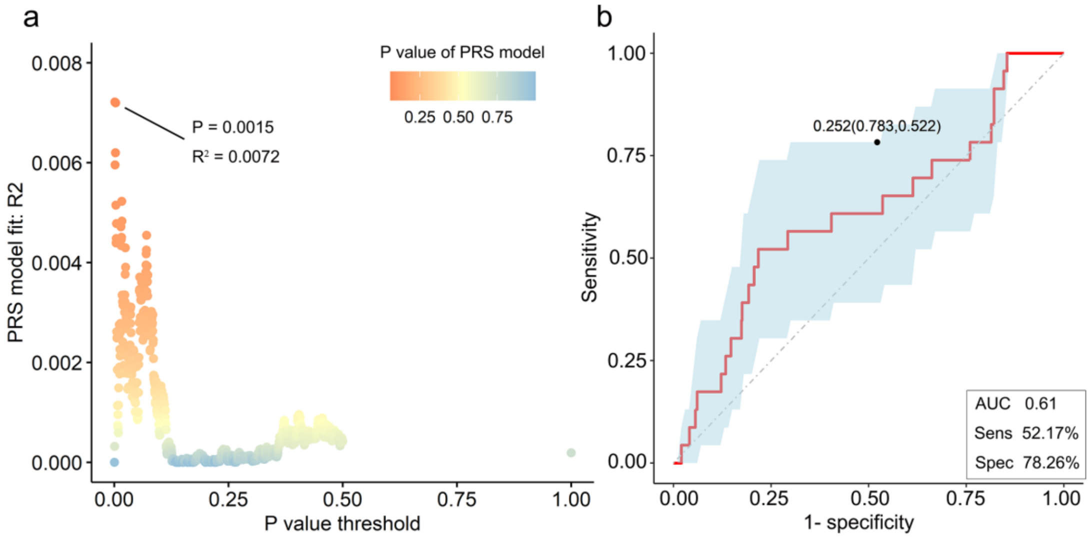

Supplementary Figure 1. PRS Nagelkerke R^2 and receiver-operating characteristic curves for TRS PRS

(a). PRS Nagelkerke R^2 for TRS. (b). The AUC is the PRS performances in the UKB cohort. AUC, area under the curve; sens, sensitivity; spec, specificity.
