# Supplementary material for: Mendelian randomization study of the relationship between blood and urine biomarkers and schizophrenia in the UK Biobank cohort
Source: Commun Med (Lond). 2024 Mar 7;4:40. doi: 10.1038/s43856-024-00467-1 (PMC10920902; doi:10.1038/s43856-024-00467-1)
Supplement: Supplementary file 2 — Description of Additional Supplementary Files [file 43856_2024_467_MOESM2_ESM.pdf]

Description of Additional Supplementary Files

Supplementary Data 1. The detailed statistical information for raw values of serum and urine biomarkers
Supplementary Data 2. The number of SNPs across the threshold the in PRS models
Supplementary Data 3. Pearson correlation analysis results between serum/urine biomarkers and TRS-PRS in the total population
Supplementary Data 4. Pearson correlation analysis results between serum/urine biomarkers and TRS-PRS in the male subpopulation
Supplementary Data 5. Pearson correlation analysis results between serum/urine biomarkers and TRS-PRS in the female subpopulation
Supplementary Data 6. MR estimates of genetic instruments for biomarkers
Supplementary Data 7. MR heterogeneity estimates (Cochrane's Q) of all genetic instruments
Supplementary Data 8. MR-Egger estimates of all genetic instruments
