# Supplementary material for: Mendelian randomization study of the relationship between blood and urine biomarkers and schizophrenia in the UK Biobank cohort
Source: Commun Med (Lond). 2024 Mar 7;4:40. doi: 10.1038/s43856-024-00467-1 (PMC10920902; doi:10.1038/s43856-024-00467-1)
Supplement: Supplementary file 11 — Reporting Summary [file 43856_2024_467_MOESM11_ESM.pdf]

nature portfolio

Corresponding author(s): DBPR COMMSMED-23-0016C

Last updated by author(s): Jan 3, 2024

Reporting Summary

Nature Portfolio wishes to improve the reproducibility of the work that we publish. This form provides structure for consistency and transparency in reporting. For further information on Nature Portfolio policies, see our Editorial Policies and the Editorial Policy Checklist.

Statistics

For all statistical analyses, confirm that the following items are present in the figure legend, table legend, main text, or Methods section.

n/a	Confirmed	
☐	☒	The exact sample size (n) for each experimental group/condition, given as a discrete number and unit of measurement
☒	☐	A statement on whether measurements were taken from distinct samples or whether the same sample was measured repeatedly
☐	☒	The statistical test(s) used AND whether they are one- or two-sided<br>*Only common tests should be described solely by name; describe more complex techniques in the Methods section.*
☐	☒	A description of all covariates tested
☐	☒	A description of any assumptions or corrections, such as tests of normality and adjustment for multiple comparisons
☐	☒	A full description of the statistical parameters including central tendency (e.g. means) or other basic estimates (e.g. regression coefficient) AND variation (e.g. standard deviation) or associated estimates of uncertainty (e.g. confidence intervals)
☐	☒	For null hypothesis testing, the test statistic (e.g. F, t, r) with confidence intervals, effect sizes, degrees of freedom and P value noted<br>*Give P values as exact values whenever suitable.*
☒	☐	For Bayesian analysis, information on the choice of priors and Markov chain Monte Carlo settings
☐	☒	For hierarchical and complex designs, identification of the appropriate level for tests and full reporting of outcomes
☐	☒	Estimates of effect sizes (e.g. Cohen's d, Pearson's r), indicating how they were calculated

Our web collection on statistics for biologists contains articles on many of the points above.

Software and code

Policy information about availability of computer code

Data collection	The UKB performed laboratory testing of commonly measured biomarkers in serum (Category 100080) and urine (Category 100083) on a cohort with extensive phenotype and genome-wide genotype data, including the unrelated individuals in this study23. Health-related records of each participant, including age and sex, were collected through either a screenshot questionnaire or verbal interview within the assessment center. The urine assays category contains information on the assays that have been performed on the UKB urine samples (https://biobank.ndph.ox.ac.uk/showcase/label.cgi?id=100083), while the blood assays category contains information on the assays that have been performed on the UKB blood samples and their results (https://biobank.ndph.ox.ac.uk/showcase/label.cgi?id=100080), including blood count (Category 100081) and blood biochemistry (Category 17518).
Data analysis	All statistical analyses, including AUC, linear regression, and Pearson correlation analyses, were performed using R software (version R 4.3.0).

For manuscripts utilizing custom algorithms or software that are central to the research but not yet described in published literature, software must be made available to editors and reviewers. We strongly encourage code deposition in a community repository (e.g. GitHub). See the Nature Portfolio guidelines for submitting code & software for further information.

Data

Policy information about availability of data

All manuscripts must include a data availability statement. This statement should provide the following information, where applicable:

- Accession codes, unique identifiers, or web links for publicly available datasets
- A description of any restrictions on data availability
- For clinical datasets or third party data, please ensure that the statement adheres to our policy

The UK Biobank data are available through the UK Biobank Access Management System at https://www.ukbiobank.ac.uk/. We will return the derived data fields following UK Biobank policy; in due course, they will be available through the UK Biobank Access Management System.

Human research participants

Policy information about studies involving human research participants and Sex and Gender in Research.

Reporting on sex and gender	The study population consisted of 202,434 male and 174,373 female subjects.
Population characteristics	This cross-sectional study included a total of 376,807 participants aged between 39 to 73 years, with a mean age of 56.99 years and a standard deviation of 7.93 years.
Recruitment	The phenotypic and genotypic data used in this study were obtained from the UKB, which conducted a large prospective cohort study from 2006 to 2010.
Ethics oversight	The present study was conducted utilizing the UKB resource, which was obtained under the application number 46478. The ethical approval of UKB was granted by the National Health Service National Research Ethics Service (reference 11/NW/0382), ensuring that the study adheres to the ethical guidelines and regulations prescribed by the regulatory authorities.

Note that full information on the approval of the study protocol must also be provided in the manuscript.

Field-specific reporting

Please select the one below that is the best fit for your research. If you are not sure, read the appropriate sections before making your selection.

☒ Life sciences ☐ Behavioural & social sciences ☐ Ecological, evolutionary & environmental sciences

For a reference copy of the document with all sections, see nature.com/documents/nr-reporting-summary-flat.pdf

Life sciences study design

All studies must disclose on these points even when the disclosure is negative.

Sample size	This cross-sectional study included a total of 376,807 participants .
Data exclusions	After removing individuals who reported inconsistencies between self-reported sex and genetic sex, as well as those with missing covariate information, 376,807 individuals of “white British ancestry subset” (UK Biobank field ID: 21000) were retained for further analysis.
Replication	Our study is a polygenic risk score analysis and Mendelian randomization study in UK Biobank cohort, and does not involve Replication.
Randomization	Our study does not involve Randomization. We employed Mendelian randomization analysis to simulate the effects of a randomized experiment.
Blinding	Our study is a polygenic risk score analysis and Mendelian randomization study in UK Biobank cohort and does not involve Blinding.

Reporting for specific materials, systems and methods

We require information from authors about some types of materials, experimental systems and methods used in many studies. Here, indicate whether each material, system or method listed is relevant to your study. If you are not sure if a list item applies to your research, read the appropriate section before selecting a response.

Materials & experimental systems

n/a	Involved in the study
☒	☐ Antibodies
☒	☐ Eukaryotic cell lines
☒	☐ Palaeontology and archaeology
☒	☐ Animals and other organisms
☒	☐ Clinical data
☒	☐ Dual use research of concern

Methods

n/a	Involved in the study
☒	☐ ChIP-seq
☒	☐ Flow cytometry
☒	☐ MRI-based neuroimaging
